# Unmodified Plant and Waste Oils as Functional Additives in PU Flooring Adhesives: A Comparative Study

**DOI:** 10.3390/molecules30183780

**Published:** 2025-09-17

**Authors:** Żaneta Ciastowicz, Renata Pamuła, Edyta Pęczek, Paweł Telega, Łukasz Bobak, Andrzej Białowiec

**Affiliations:** 1 Department of Applied Bioeconomy, Wrocław University of Environmental and Life Sciences, 37a Chełmońskiego Str., 51-630 Wroclaw, Poland; zaneta.ciastowicz@upwr.edu.pl (Ż.C.); edyta.peczek@upwr.edu.pl (E.P.); pawel.telega@upwr.edu.pl (P.T.); 2 Selena Industrial Technologies Sp. z o.o., Pieszycka 3, 58-200 Dzierzoniow, Poland; renata.pamula@selena.com; 3 Department of Functional Food Products Development, Wrocław University of Environmental and Life Sciences, 51-630 Wroclaw, Poland; lukasz.bobak@upwr.edu.pl

**Keywords:** two-component polyurethane adhesive, castor oil, rapeseed oil, sunflower oil, linseed oil, used cooking oil, VOC screening, leaching, parquet flooring

## Abstract

This work compares reactive (castor) and non-reactive (rapeseed, sunflower, linseed, and used cooking) oils, each at a dosage of 10 wt%, when incorporated into an in-house two-component polyurethane (PU) parquet adhesive. A commercial market adhesive was tested only as an external benchmark and was not modified. Mechanical properties were evaluated according to EN ISO 17178, inorganic leaching according to EN 12457-4, and volatile organics were screened by headspace GC–MS (not comparable to ISO 16000-9 chamber protocols). All in-house formulations met the EN ISO 17178 acceptance limits. The sunflower oil variant showed the highest shear strength, whereas rapeseed and castor oils provided stable tensile performance. HS-GC-MS did not yield high-confidence VOC identifications; therefore, no regulatory emission claim is made. The formulation with used cooking oil exhibited the largest variability and elevated leaching of Zn and Sb, underscoring the need for feedstock quality control. At 10 wt% loading, standard-compliant performance was obtained with both reactive and non-reactive oils, suggesting that physical modification can be sufficient, while castor oil may additionally react. In contrast to derivatized oils reported elsewhere, our approach employs unmodified oils, thereby avoiding extra reaction steps—such as epoxidation, hydroxylation, and transesterification—that typically increase the carbon footprint, while still meeting relevant standards. Full VOC chamber testing and LCA are beyond the scope of this study.

## 1. Introduction

Polyurethane (PU) adhesives are extensively used in the construction industry due to their strong bonding performance, versatility, and ease of application. In parquet flooring systems, two-component PU adhesives represent a standard solution, offering excellent mechanical properties and moisture resistance. However, conventional formulations are primarily based on petrochemical-derived raw materials and may contribute to the emission of volatile organic compounds (VOCs), potentially compromising indoor air quality and posing environmental and health-related risks [1].

In recent years, regulatory developments within the European Union (EU) have increasingly addressed emissions from construction products, particularly VOCs. The Construction Products Regulation (EU) No 305/2011 (CPR) establishes harmonized conditions for the marketing of construction products across the EU, focusing on essential characteristics such as mechanical strength, fire safety, and hygiene. However, the CPR does not set specific VOC emission limits, allowing Member States to introduce additional national requirements concerning health and environmental protection [2].

As a result, countries such as Germany and France have adopted more stringent national regulatory frameworks. Germany applies the AgBB evaluation scheme, while France enforces mandatory VOC labeling for construction materials. These national schemes often exceed the harmonized EU requirements and are widely used to guide product approvals for indoor applications [3,4].

Within this regulatory context, there is growing interest in developing sustainable adhesive formulations that not only comply with VOC emission thresholds but also retain adequate mechanical performance. One promising approach involves the partial substitution of petrochemical-based ingredients with renewable raw materials, such as vegetable oils and waste oils [5,6]. Previous research has explored the use of bio-based polyols and plasticizers in PU systems [7]. In contrast to that body of work, we deliberately study unmodified vegetable and waste oils as functional modifiers in 2K parquet PU adhesives. This avoids additional processing steps (epoxidation, hydroxylation, transesterification, esterification) that generally add energy and reagent demand—and thus embodied carbon—while enabling a direct, application-level assessment However, the direct use of unmodified vegetable and waste oils in parquet adhesives remains insufficiently studied—particularly concerning VOC emissions, long-term mechanical performance, and the environmental safety of the cured products.

This study evaluates in-house two-component PU parquet adhesives modified with 10 wt% of reactive (castor) and non-reactive (rapeseed, sunflower, linseed, and used cooking) oils. A commercial PU adhesive is included solely as an external benchmark. Mechanical properties were evaluated according to EN ISO 17178 [8] (including thermal aging), inorganic leaching according to EN 12457-4 [9], and volatile organics were screened by headspace GC–MS (not comparable to ISO 16000-9 [10] chamber protocols). The objective is to determine whether standard-compliant performance can be achieved at a practical loading level without chemical incorporation for non-reactive oils, while clearly stating the limitations related to VOC assessment and environmental claims. To address structure property relationships, we compile chemical descriptors of the oils and relate them to PU performance (Table 1; SI Tables S1 and S2).

## 2. Results

### 2.1. Structure of the Oils and Structure–Property Relationships

To rationalize the performance trends reported later, we first compare the physicochemical parameters of the five oils used in this work—acid value (AV), hydroxyl value (OHV), iodine value (IV), peroxide value (PV), water content, viscosity, density, and fatty-acid (FA) profiles—compiled from our previous study on these very oils [11].

Summary values are reported in Table 1; full fatty acid distributions are provided in the Supplementary Information (Tables S1 and S2; adapted from [11]). Representative molecular structures are shown in Figure S1 (SI) to visualize the presence (castor; ricinoleic 12-OH) vs. absence (rapeseed, sunflower, linseed) of pendant –OH groups; UCO is a variable mixture. Reaction level schematics are provided in Scheme S1 (SI), illustrating urethane formation between –NCO and –OH (castor oil) and the moisture side reaction to urea and CO_2_.

Castor oil is distinguished by a very high OHV (158 mg KOH g^−1^) arising from 85 wt% ricinoleic acid, enabling covalent incorporation into the polyurethane network (–NCO/–OH). Accordingly, castor oil is expected to provide higher cohesion and reduced migration/leachability relative to non-reactive oils. Its comparatively high neat oil viscosity can also increase the formulation viscosity.

By contrast, rapeseed, sunflower, and linseed oils exhibit near-zero OHV (≤~4 mg KOH g^−1^) and therefore behave predominantly as non-reactive plasticizers; among them, linseed shows the highest IV (173 g I_2_ 100 g^−1^), indicating a highly unsaturated, oxidation-prone profile.

Used cooking oil (UCO) remains largely non-reactive by OHV (4.2 mg KOH g^−1^) but typically presents elevated PV, AV, and moisture, reflecting prior thermal/oxidative history and batch variability; these features can promote side reactions with isocyanates and disturb cure kinetics.

In summary, reactive vs. non-reactive character (OHV) and degree of unsaturation (IV) are the primary axes controlling oil behavior in 1K moisture-curing PU adhesives: reactive, OH rich oils (castor) can become part of the network; low OHV oils act mainly as physical plasticizers, with higher IV introducing oxidative sensitivity (linseed) and UCO adding variability via PV, AV, water content. This structure-led framework underpins the interpretations offered in the following sections on viscosity (Section 2.2), mechanical performance (Section 2.4), and leaching, VOC (Section 2.3 and Section 2.5), and is visualized in the qualitative structure–property map.

### 2.2. Rheological Analysis

Given the large spread in OHV, IV and neat oil viscosity summarized in Table 1 (Section 2.1), we anticipated that castor oil (high OHV, high neat viscosity) would raise formulation viscosity, whereas low OHV oils (rapeseed, sunflower, linseed) would primarily act as plasticizers and lower it, with UCO falling in between due to its higher neat viscosity and oxidation products. Indeed, the measured viscosity values follow this pattern at the test temperature and shear rate. This trend is consistent also with Scheme S1: castor’s pendant –OH can react (panel A), whereas low OHV oils act mainly as non-reactive plasticizers; moisture-driven consumption of –NCO is depicted in panel B.

The viscosity of the polyurethane formulations was significantly influenced by the type of oil-based modifier used.

**Figure 1:**
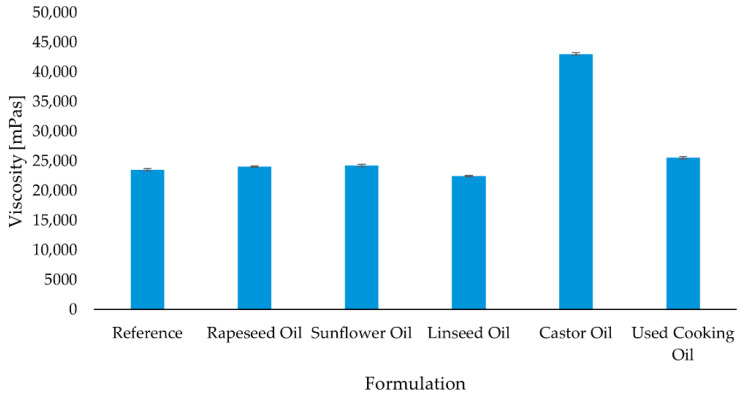
Average viscosity of formulations modified with different oils. Error bars represent the standard error of the mean (SEM), calculated from five replicates (n = 5).

As shown in Figure 1, the formulation containing castor oil exhibited the highest average viscosity (43,060 mPas), suggesting a strong thickening effect due to the high polarity and hydroxyl group content of the castor oil [5,6]. In contrast, the linseed oil formulation showed the lowest viscosity (22,520 mPas), slightly below that of the unmodified reference system (23,560 mPas). Formulations modified with rapeseed oil, sunflower oil, and used cooking oil displayed intermediate viscosity values, ranging from 24,080 mPas to 25,720 mPas. Among these, the adhesive based on used cooking oil showed a moderate increase in viscosity compared to the reference, potentially due to the presence of thermally altered or oxidized compounds that influence rheological behavior. Overall, the data indicate that both the chemical structure and processing history of the incorporated oil phase affect the viscosity of the final adhesive system. In addition to the chemical composition and polarity of the oil modifiers, the intrinsic viscosity of the oils themselves contributes to the overall viscosity of the formulations. This is primarily a physical effect related to the volumetric contribution of the liquid phase. Oils with inherently higher viscosities—such as castor oil and used cooking oil—tend to increase the viscosity of the adhesive mixture more significantly than low-viscosity vegetable oils. Notably, used cooking oil, which undergoes oxidative and thermal degradation during use, often exhibits elevated viscosity due to the presence of higher-molecular-weight and polymerized compounds [11].

### 2.3. VOC Screening (HS-GC–MS)

**Figure 2:**
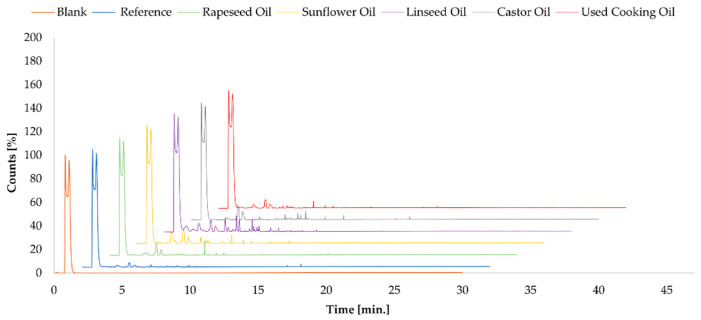
Overlaid total ion chromatograms (TICs) from headspace GC–MS analysis of adhesive samples. Major peaks corresponding to identifiable VOCs are marked. The signal at RT ≈ 0.8–1.1 min, corresponding to nitrous oxide, is visible across most samples and was identified as a non-sample-related artifact.

Based on the structure property framework (Section 2.1; Table 1), we anticipated that non-reactive, low-OHV oils could release more light headspace actives (higher propensity to migrate, volatilize), whereas castor oil, capable of covalent incorporation, would show reduced headspace signals. Headspace GC–MS of the cured polyurethane adhesives was used as a qualitative screening tool (not comparable to EN 16516/AgBB chamber protocols; no TVOC or compliance claims are made). Total ion chromatograms (TICs) for each formulation are shown in Figure 2 and illustrate differences in the headspace profiles of the bio-oil variants. For tentative assignments, only peaks with a library match > 750 were considered; such assignments should be regarded as indicative. The sunflower oil-based formulation exhibited two distinct VOC peaks at retention times of 3.531 min and 4.843 min, identified as 1-cycloocten-5-yne and a diphosphoric ester, respectively. Both compounds are considered to be of low toxicological concern and are not listed as Substances of Very High Concern (SVHC) under the REACH regulation. They are also not classified as carcinogenic, mutagenic, or reprotoxic (CMR). The linseed oil formulation displayed the most complex VOC profile among all tested samples. Detected compounds included 1-cycloocten-5-yne (7.1%), 3,4-octadiene-7-methyl (4.9%), diisooctyl phosphate ester (4.5%), and a nitrogen-containing heterocycle, diazabicycloheptene (2.9%). These results suggest partial degradation or volatilization of unsaturated oil components under thermal conditioning. None of the identified compounds is currently listed as SVHC under REACH, nor are they classified as CMR substances. Although some compounds—such as phosphate esters and heterocycles—may raise toxicological concerns in other contexts, their presence at low concentrations and lack of formal classification indicate a low regulatory and health risk under the tested conditions. The sample modified with used cooking oil did not yield any identifiable organic VOCs above the confidence threshold, indicating either chemical inertness or suppression of volatile release. Similarly, the castor oil-based formulation showed no identifiable VOC peaks above the threshold score, which may reflect strong matrix binding or high thermal stability of the incorporated species. No additional organic volatiles were detected in the reference sample or in the rapeseed oil formulation, confirming the overall low-emission profile of these adhesive systems under the applied test conditions. Overall, the HS-GC–MS data are used here solely to compare formulations qualitatively; they are not suitable for hazard assessment or regulatory interpretation.

### 2.4. Mechanical Properties

In light of the structure and property framework (Section 2.1; Table 1) and the rheology trends (Section 2.2), we anticipated that castor oil (high OHV, high neat viscosity) could enhance mechanical properties via chemical incorporation into the PU network, whereas low OHV oils (rapeseed, sunflower, linseed) would mostly act as non-reactive plasticizers, improving wetting and stress redistribution but not contributing covalent links; UCO was expected to underperform due to elevated PV, AV, moisture and side reactions. The mechanical data broadly align with this picture, while also revealing that interfacial effects (wetting and penetration) can outweigh bulk-network effects at the studied loading, particularly for sunflower and rapeseed.

#### 2.4.1. Shear Strength Properties

**Figure 3:**
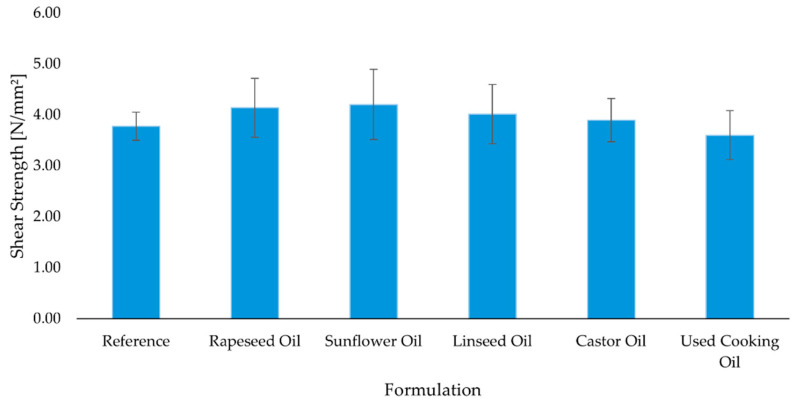
Mean shear strength of polyurethane adhesives after 3 days of curing (n = 10). Error bars represent ± standard deviation.

**Figure 4:**
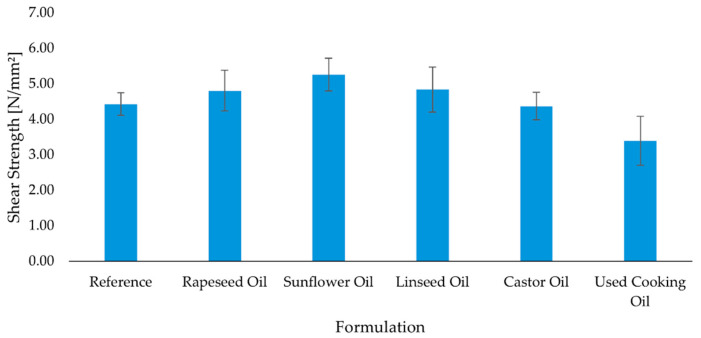
Mean shear strength of polyurethane adhesives after thermal aging (n = 10). Error bars represent ± standard deviation.

Consistent with Section 2.1 and Section 2.2, low OHV, low viscosity sunflower and rapeseed oils delivered the highest shear strengths at 3 days and after conditioning (Figure 3 and Figure 4), which we attribute to superior wetting, penetration into the wood substrate and balanced stiffness of the adhesive layer. Castor oil, despite its capacity for covalent incorporation, produced comparable but not superior shear values, likely because its higher formulation viscosity (Section 2.2) limited interfacial wetting, partly offsetting the network benefit. Linseed remained competitive at 3 days but did not lead after conditioning, consistent with its high IV and greater oxidative susceptibility. UCO underperformed and even declined after thermal conditioning, in line with oxidation products, moisture-induced side reactions that can create microvoids or embrittlement.

All adhesive formulations met the classification criteria for hard elastic adhesives, as defined by EN ISO 17178. Shear strength values were evaluated after 3 days of standard curing and after 28 days of combined standard and accelerated thermal conditioning (Figure 3 and Figure 4).

After 3 days, the reference adhesive exhibited a mean shear strength of 3.78 ± 0.28 N/mm^2^. All bio-based formulations demonstrated comparable or improved performance. The highest value was recorded for the sunflower oil-based adhesive (4.20 ± 0.69 N/mm^2^), followed by the rapeseed oil (4.14 ± 0.58 N/mm^2^) and linseed oil (4.01 ± 0.58 N/mm^2^) formulations. The adhesives based on castor oil (3.89 ± 0.42 N/mm^2^) and used cooking oil (3.60 ± 0.48 N/mm^2^) showed slightly lower, but still satisfactory, values (Figure 3). After 28 days, most formulations showed further increases in shear strength, indicative of continued crosslinking and structural consolidation. The sunflower oil-based formulation remained the best-performing system (5.26 ± 0.46 N/mm^2^), followed by linseed oil (4.84 ± 0.64 N/mm^2^) and rapeseed oil (4.80 ± 0.57 N/mm^2^). The reference adhesive increased to 4.43 ± 0.32 N/mm^2^, confirming the beneficial effect of prolonged curing. In contrast, the formulation with used cooking oil decreased slightly (3.39 ± 0.69 N/mm^2^), potentially due to slower reaction kinetics or partial degradation under thermal stress (Figure 4). These findings support the technical feasibility of partially replacing petrochemical components in polyurethane adhesives with unmodified vegetable or waste oils—particularly sunflower and rapeseed oils—without compromising mechanical integrity.

#### 2.4.2. Tensile Strength Properties

**Figure 5:**
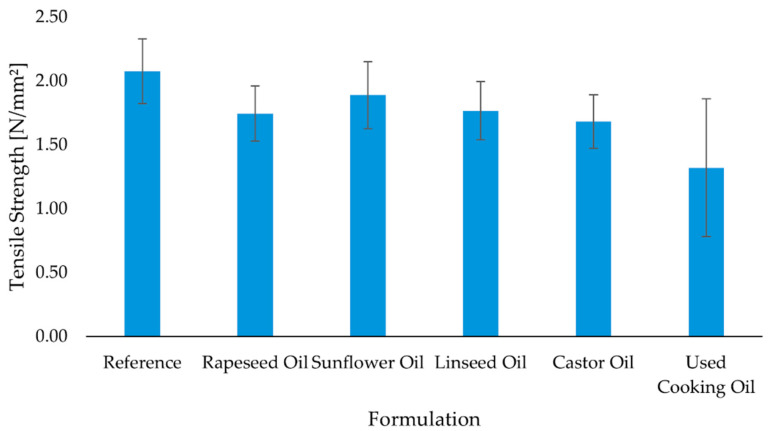
Mean tensile strength of polyurethane adhesives after 7 days of curing (n = 10). Error bars represent ± standard deviation.

**Figure 6:**
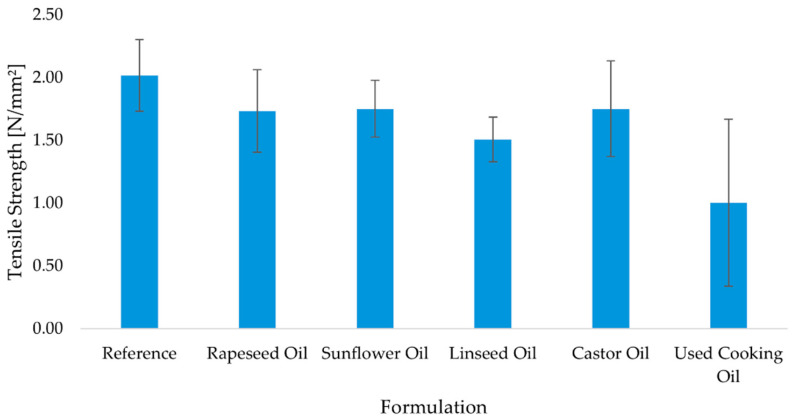
Mean tensile strength of polyurethane adhesives after 28 days of conditioning (n = 10). Error bars represent ± standard deviation.

**Figure 7:**
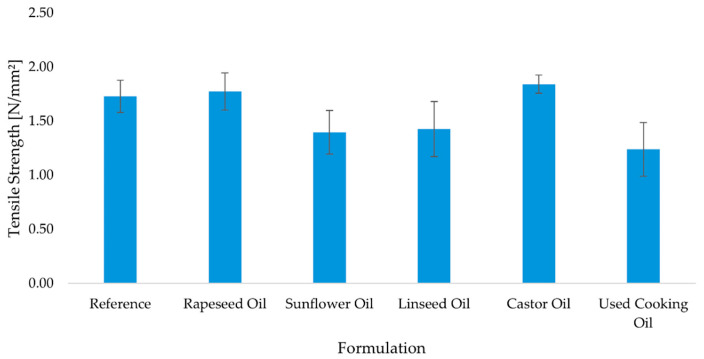
Mean tensile strength of polyurethane adhesives after thermal aging (n = 10). Error bars represent ± standard deviation.

Tensile testing reproduces the same qualitative picture with nuanced time-dependence (Figure 5, Figure 6 and Figure 7). The reference formulation led at 7 days, indicating rapid interfacial cohesion, while sunflower oil and rapeseed oil remained competitive among bio-modified systems. Castor oil tracked close to the leaders across time points (including after aging), consistent with a gradual network build-up that compensates its initial wetting penalty. Linseed drifted lower after prolonged conditioning, in line with its higher unsaturation (IV) and oxidative sensitivity. UCO again showed the lowest mean and highest scatter, pointing to variable curing and degradation pathways.

Tensile strength was assessed after 7 days of standard curing, after 28 days of curing, and following thermal aging, in order to evaluate both short-term and long-term bonding performance of the adhesive formulations under conditions simulating real-world exposure. Results are presented in Figure 5, Figure 6 and Figure 7.

After 7 days of curing, the reference adhesive exhibited the highest tensile strength (2.08 ± 0.25 N/mm^2^), indicating rapid development of interfacial cohesion. Among the bio-based adhesives, the sunflower oil (1.89 ± 0.26 N/mm^2^) and rapeseed oil (1.74 ± 0.22 N/mm^2^) formulations showed competitive values, while castor oil (1.68 ± 0.21 N/mm^2^) and linseed oil (1.77 ± 0.23 N/mm^2^) performed slightly lower. The used cooking oil formulation again demonstrated the weakest bonding, with a mean value of 1.32 ± 0.54 N/mm^2^ (Figure 5). After 28 days of standard curing, a modest decrease in tensile strength was observed in most formulations. The reference adhesive declined slightly to 1.73 ± 0.15 N/mm^2^, while the castor oil and rapeseed oil formulations remained relatively stable (1.84 ± 0.08 N/mm^2^ and 1.77 ± 0.17 N/mm^2^, respectively). By contrast, the sunflower oil (1.40 ± 0.20 N/mm^2^), linseed oil (1.43 ± 0.25 N/mm^2^), and used cooking oil (1.24 ± 0.25 N/mm^2^) formulations showed reduced adhesion (Figure 7). Following the aging protocol, which involved extended conditioning at elevated temperature, further differentiation between formulations was observed. The reference adhesive recovered to 2.02 ± 0.28 N/mm^2^, and the sunflower oil formulation stabilized at 1.75 ± 0.23 N/mm^2^, matching the performance of castor oil (1.75 ± 0.38 N/mm^2^). Rapeseed oil performed slightly lower (1.73 ± 0.33 N/mm^2^), while linseed oil declined to 1.51 ± 0.18 N/mm^2^. The used cooking oil adhesive exhibited the lowest strength and the highest variability (1.00 ± 0.67 N/mm^2^), indicating susceptibility to thermal degradation (Figure 6). In summary, the results demonstrate that several bio-based adhesive systems—particularly those modified with rapeseed, sunflower, and castor oil—maintain satisfactory tensile strength after prolonged curing and thermal aging. However, formulations containing used cooking oil show reduced mechanical stability and greater variability, suggesting potential limitations in long-term durability.

### 2.5. Leaching Behavior

Within the structure property framework (Section 2.1; Table 1) and in line with the VOC screening (Section 2.3), we expected that non-reactive, low OHV oils would retain a larger water extractable organic fraction—quantified here as dissolved organic carbon (DOC), whereas castor oil, capable of covalent incorporation, would show reduced leachability. We also anticipated that UCO, due to its oxidative properties and thermal history (elevated PV, AV, degradation products), could yield higher DOC and greater variability. The results are consistent with this picture: metals and major anions were below reporting limits across all systems, while DOC differentiated formulations with UCO showing the highest values and castor among the lowest, indicating that oil reactivity and processing history govern the small residual, water-extractable organic fraction, rather than inorganic release.

Leaching tests conducted on all adhesive formulations revealed low environmental mobility of the analyzed elements and anions. Most tested parameters were below the respective reporting limits in all samples, indicating minimal potential for the release of hazardous substances under the applied extraction conditions.

**Figure 8:**
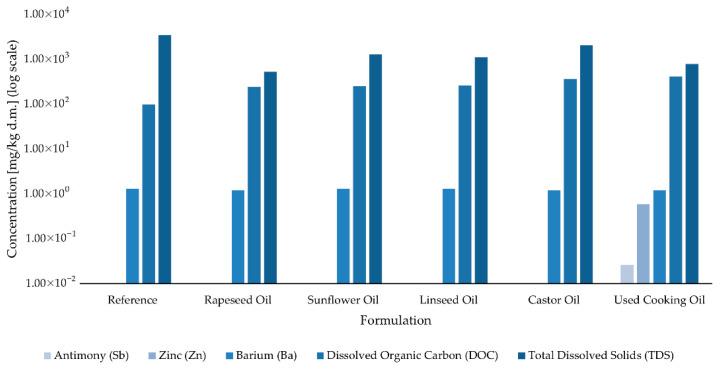
Leaching levels of antimony (Sb), zinc (Zn), barium (Ba), dissolved organic carbon (DOC), and total dissolved solids (TDS) from adhesive formulations.

Table 2 summarizes the quantitative results for heavy metals and selected inorganic anions. Most metals, including mercury (Hg), molybdenum (Mo), nickel (Ni), lead (Pb), selenium (Se), copper (Cu), arsenic (As), chromium (Cr), and cadmium (Cd)—were found to be below the quantification threshold in all tested adhesives. Similarly, chlorides (Cl^−^), fluorides (F^−^), and sulfates (SO_4_^2−^) were not detected above their reporting limits in any formulation. Despite the overall low leaching levels, a few differences between formulations were observed. In the sample modified with used cooking oil, zinc (Zn) and antimony (Sb) were the only elements detected above the quantification limit, with concentrations of 0.58 mg/kg d.m. and 0.026 mg/kg d.m., respectively. In all other formulations, Zn and Sb remained below the detection threshold. Barium (Ba) was consistently present in all samples, with concentrations ranging from 1.2 to 1.3 mg/kg d.m., suggesting that its release was independent of the type of oil used in the adhesive formulation. In contrast, dissolved organic carbon (DOC) and total dissolved solids (TDS) showed greater variation across formulations. DOC ranged from 98 mg/kg d.m. in the reference sample to 410 mg/kg d.m. in the sample containing used cooking oil. TDS values ranged from 520 to 3430 mg/kg d.m., with the highest level again observed in the reference adhesive. These findings are graphically illustrated in Figure 8, which compares the leaching levels of Sb, Zn, Ba, DOC, and TDS across all formulations using a logarithmic scale.

All tested formulations exhibited leaching values that meet the threshold limits for inert waste specified in Council Directive 1999/31/EC and Decision 2003/33/EC. These results are consistent with previous studies on polyurethane-based systems, which have demonstrated low leaching potential for both inorganic and organic components. In particular, polyurethane coatings and membranes have been shown to release only trace amounts of zinc and dissolved organic carbon, with concentrations well below the threshold limits set by the European Directive [12].

### 2.6. Calorimetric Results

The calorific value of the cured adhesive formulations was evaluated using bomb calorimetry and is presented in Table 3. All bio-based adhesives exhibited slightly higher heating values (HHVs) compared to the reference formulation, which did not contain any renewable oil additives. The formulation modified with sunflower oil demonstrated the highest HHV (16,120 J/g), followed by those containing rapeseed oil (15,898 J/g) and linseed oil (15,895 J/g). The castor oil and used cooking oil variants showed intermediate values (15,830 J/g and 15,876 J/g, respectively), while the reference formulation exhibited the lowest HHV (15,803 J/g). These differences, although modest, may reflect the higher organic content and degree of unsaturation associated with the incorporated vegetable oils. Although many studies have investigated the thermal degradation of polyurethane foams derived from vegetable oils, there is limited data on the calorific value (HHV) of cured bio-based polyurethane adhesives. The results presented in this study provide new insights that may be relevant for future research on energy recovery at the end-of-life stage, such as in life cycle assessment (LCA).

## 3. Discussion

The results of this study confirm that unmodified vegetable and waste oils can be effectively incorporated into two-component polyurethane (PU) adhesives for parquet applications as partial substitutes for petrochemical ingredients. The inclusion of 10 wt% bio-based oil modifiers introduced distinct and measurable effects on rheological behavior, headspace profiles (HS-GC–MS screening), mechanical performance, leaching characteristics, and calorific value—without compromising the essential functional properties required for parquet bonding. These changes reflect both the intrinsic chemical composition and physical properties of the incorporated oils, as well as their interactions with the PU matrix. From a processing standpoint, the observed differences in viscosity among the modified formulations underscore the role of both molecular polarity and intrinsic viscosity of the oils. Castor oil, characterized by a high hydroxyl content, increased the viscosity of the adhesive, which may affect flow and application behavior. Similarly, used cooking oil (UCO) also elevated viscosity, likely due to thermally altered, partially polymerized species that increase effective molecular weight and reduce flowability. In contrast, linseed oil decreased overall viscosity below that of the reference formulation, highlighting the potential to tailor application properties through careful selection of oils based on polarity and chain-length distribution [11]. These viscosity trends thickening by castor and dilution by low-OHV triglycerides are consistent with prior observations for castor oil-based polyols versus non-reactive oils in PU systems [5,6,7].

Headspace GC–MS was applied as a qualitative screening tool (not comparable to EN 16516/AgBB chamber protocols; no TVOC values or compliance claims are made). Within this screening, differences in headspace profiles were observed and are attributable, in first approximation, to the chemical nature of the incorporated oils. As all samples were prepared from identical polyol, isocyanate and fillers under the same conditions, the oil type was the primary varying factor; however, typical headspace artifacts and matrix effects cannot be fully excluded. The linseed- and sunflower-oil systems produced peaks with tentative library assignments (match > 750). In the linseed oil formulation, several candidate compounds were indicated (unsaturated hydrocarbons, phosphate esters, a nitrogen-containing heterocycle), whereas the sunflower oil showed a simpler profile with two main tentative components. These signals may originate from naturally occurring constituents or pre-existing oxidation products of unsaturated fatty acids under the applied headspace conditions, but they do not permit firm structural or quantitative conclusions. Based on these tentative assignments and a desk check of public hazard classifications, none of the candidate structures is classified as CMR (Categories 1A/1B) under CLP nor present on the REACH Candidate List of SVHC. Nevertheless, they would contribute to chamber-derived TVOC and could influence compliance in schemes such as AgBB or Émissions dans l’air intérieur, which evaluate total emissions irrespective of toxicological classification. Adhesives based on rapeseed oil, castor oil, and UCO displayed no peaks above the identification threshold (match > 750); this does not imply the absence of emissions, only the lack of high-confidence library matches in this screening. Given that PU parquet adhesives are often used with underfloor heating (up to ~50–55 °C), complementary dynamic or chamber-based testing (e.g., ISO 16000-9 [10]) is recommended for low-emission certification and indoor-air assessments. Mechanical testing confirmed that all adhesive systems met the performance thresholds specified by EN ISO 17178 for hard elastic adhesives. Among the modified systems, sunflower and rapeseed oil-based adhesives offered the most favorable balance between early strength development and long-term durability. These systems retained high shear and tensile strength even after thermal aging, indicating good compatibility and crosslinking efficiency with the PU matrix. In contrast, UCO-based adhesives exhibited reduced mechanical stability and greater variability—particularly in tensile strength after aging—likely due to inconsistent feedstock quality and oxidative degradation by-products. Maintaining EN ISO 17178-compliant shear and tensile performance at a practical 10 wt% loading aligns with reports where modest bio-oil additions preserved bond integrity [7,13].

From an environmental perspective, all tested adhesives complied with the criteria for inert waste landfill acceptance under Council Directive 1999/31/EC and Decision 2003/33/EC. The low levels of heavy metals and anions in leachates confirm the environmental safety of the cured systems. Slightly elevated DOC and Zn levels in the UCO-based formulation suggest residual variability due to the heterogeneous nature of this feedstock, reinforcing the need for upstream quality control when using recycled oils, especially in eco-labeled or low-emission products. The overall low inorganic leaching agrees with studies on cured PU coatings, membranes reporting only trace releases under extraction protocols [12,14]. The elevated DOC in the UCO variant is compatible with moisture and oxidation-driven side reactions that divert –NCO to urea formation (Scheme S1B), leaving more water-extractable organics, as also noted for oxidized feeds in prior work [11].

Additionally, calorimetric analysis showed slightly higher heating values (HHVs) for bio-based adhesives compared to the reference. The highest HHV was recorded for the sunflower oil formulation, reflecting increased energy content—potentially beneficial for thermal recycling. While HHV data were not the primary focus, they provide relevant input for life cycle assessment (LCA), particularly at the end-of-life stage.

In summary, this study demonstrates the technical feasibility of incorporating unmodified renewable oils into PU adhesive systems without compromising performance or environmental safety. This approach contributes to broader sustainability goals by reducing fossil resource use and minimizing VOC emissions. However, UCO incorporation requires further optimization, including potential pre-treatment or blending strategies to ensure mechanical reliability. Future research should address formulation robustness, explore hybrid oil systems, and expand environmental assessments under realistic service conditions.

## 4. Material and Methods

### 4.1. Materials

Two-component (PU) parquet adhesives were prepared using bio-based formulations containing a renewable oil additive. In the modified formulations, vegetable or used cooking-derived oils were incorporated into the adhesive composition at 10 wt% as liquid fillers.

The reference adhesive was a commercially available PU parquet system not labeled or marketed as bio-based (Selena: Artelit PB 890, Wrocław, Poland). The oils used for modification included rapeseed oil, linseed oil, sunflower oil, and used cooking oil (UCO). All oils were sourced from the following suppliers: Brenntag (Kędzierzyn-Koźle, Poland), Oquema (Ozorków, Poland), Standard (Lublin, Poland), and Euro Eko Poland (Mielec, Poland), respectively. No chemical modification of the oils was performed before use.

### 4.2. Preparation of Adhesive Samples

The adhesive formulation was prepared by simultaneously blending all components of Component A—polyol, oil additive, and mineral fillers—using a mechanical stirrer (IKA RW 16 basic, Staufen, Germany) at 500 rpm for 45 min at 23 °C. Before mixing, the mineral fillers were oven-dried at 105 °C for 24 h to ensure moisture removal and to prevent undesired side reactions during curing. The hardener (Component B), diphenylmethane diisocyanate (pMDI), was then added and mixed for an additional 2 min. The adhesive was subsequently degassed under vacuum and applied immediately.

### 4.3. Analytical Methods

#### 4.3.1. Viscosity

Viscosity was measured using a Brookfield CAP 2000+ rheometer (AMETEK Brookfield, Middleboro, MA, USA) following EN ISO 3219-2:2021 [15], employing a cone-plate configuration with spindle S05 at a temperature of 23 °C [15].

#### 4.3.2. VOC Emission Testing

Samples were analyzed using a gas chromatography–mass spectrometry (GC–MS) system (Agilent 7890B GC coupled with a 5977B MSD, Santa Clara, CA, USA) equipped with a DB-5MS capillary column (30 m × 0.25 mm i.d., 0.25 μm film thickness; Agilent Technologies, Santa Clara, CA, USA). The headspace of each sample was collected using a heated syringe (140 °C) integrated into a Gerstel MPS robotic autosampler (Mülheim an der Ruhr, Germany). Approximately 200 mg of each composite sample was placed in a 20 mL glass vial sealed with a PTFE-lined cap and incubated at 120 °C for 60 min. Subsequently, 2500 μL of the headspace gas was withdrawn and injected into the GC–MS system. The injector temperature was set to 250 °C and operated in split mode (10:1). Helium was used as the carrier gas at a constant flow rate of 1.0 mL/min. The oven temperature program was as follows: initial temperature 50 °C (held for 1 min), ramped at 10 °C/min to a final temperature of 240 °C. The quadrupole, ion source, and transfer line temperatures were maintained at 150 °C, 230 °C, and 250 °C, respectively. Compound identification was performed by comparing the obtained mass spectra with entries from the NIST17 mass spectral library.

However, all acquired spectra showed low similarity scores (<75%), which precluded confident identification of volatile organic compounds.

#### 4.3.3. Mechanical Testing

The performance of the adhesive was evaluated by means of shear and tensile strength tests, conducted following ISO 17178. All measurements were carried out using an Instron 3367 universal testing machine (Norwood, MA, USA) equipped with a 30 kN load cell [8].

##### Shear Strength—Hard Elastic Adhesives

Oak mosaic parquet fingers (160 × 23 × 8 mm) were bonded using a 1.0 ± 0.3 mm adhesive layer, applied with the aid of a custom spacer template to ensure a bonding area of 600 ± 20 mm^2^. Specimens were conditioned under two separate procedures:

‑ 3 days at 23 ± 2 °C/50 ± 5% RH,
‑ 28 days: 7 days under standard conditions, followed by 20 days at 40 ± 2 °C, and 1 additional day under standard conditions.

The samples were subjected to tensile loading at a rate of 20 ± 2 mm/min until failure occurred. Shear strength (TS) was calculated according to the following equations:

TS = F*_max_*/A (N/mm^2^)

where

*F_max_* is the maximum applied load,

*A* is the bonded area,

##### Tensile Strength

Wood blocks (50 × 50 × 10 mm) were bonded to EN 1323 [16] concrete slabs using a notched trowel. After placement, 2 kg weights were applied for 60 s to ensure proper contact. The following storage conditions were used:

‑ 7 days under standard conditions,
‑ 28 days under standard conditions,
‑ 7 days standard + 20 days at 40 °C + 1 day standard (heat aging).

Pull-head plates were bonded to the wood blocks using epoxy resin 24 h prior to testing. The load was applied at a rate of 250 ± 50 N/s until failure occurred. Tensile strength (TT) was calculated according to the formula:

TT = F*_max_*/A (N/mm^2^)

where

*F_max_* is the maximum force at failure,

*A* is the bonded area.

#### 4.3.4. Leaching Tests of Cured Adhesives

Leaching tests were conducted according to the procedure described in EN 12457-4:2004. A single-step batch extraction was carried out under static conditions using deionized water at a liquid-to-solid ratio of 10:1 (L/kg). Samples were agitated for 6 h using intensive mechanical stirring, followed by an 18 h rest period, resulting in a total extraction time of 24 h. After extraction, the leachates were filtered through 0.45 µm paper filters and stored at 4 °C until analysis. The aqueous extracts were analyzed for selected heavy metals and other pollutant indicators, including Ba, Cd, Cr, Cu, Hg, Mo, Ni, Pb, Zn, Se, Sb, As, Cl^−^, F^−^, SO_4_^2−^, dissolved organic carbon (DOC), and total dissolved solids (TDS) [9].

Quantitative analysis of Zn, Cu, Ba, Pb, Ni, Mo, Cr, and Cd was performed using inductively coupled plasma optical emission spectrometry (ICP-OES; Perkin Elmer Optima 7300 DV, Shelton, CT, USA), following ISO 11885:2009 [17].

Arsenic, selenium, and antimony were measured using inductively coupled plasma mass spectrometry (ICP-MS; Perkin Elmer NexION 2000), following EN 17294-2:2016-11 [18]. Mercury content was determined by cold vapor atomic absorption spectrometry (AAS-CV; Perkin Elmer PinAAcle 900T), in compliance with ISO 12846:2012 [19].

#### 4.3.5. Calorimetric Analysis

The higher heating value (HHV) of the cured adhesive formulations was determined following ISO 1928:2009 [20], using an IKA C200 bomb calorimeter (IKA-Werke GmbH & Co. KG, Staufen, Germany), equipped with a C248 oxygen charging station and a C5010 combustion bomb. Approximately 0.3 g of each fully cured adhesive sample was weighed into a combustion crucible and placed inside the bomb, which was then filled with oxygen to a pressure of 30 bar. Combustion was initiated electrically, and the resulting temperature rise in the surrounding water jacket was recorded. Benzoic acid was used as the calibration standard. All measurements were performed in triplicate at ambient temperature (25 ± 1 °C), and the average HHVs are reported in joules per gram (J/g) [20].

### 4.4. Statistical Analysis

Descriptive statistical analysis was performed using Microsoft Excel (Microsoft 365, Redmond, WA, USA). Data are reported as mean values accompanied by either standard deviation (SD) or standard error of the mean (SEM), as specified in the corresponding figure legends. No inferential statistical tests (e.g., ANOVA or *t*-tests) were applied. The statistical summary was intended to illustrate data dispersion and facilitate visual comparisons among the tested formulations.

## 5. Conclusions

This study demonstrates the technical viability of incorporating unmodified vegetable and waste oils into two-component polyurethane adhesives for parquet applications. All tested formulations met the mechanical performance criteria of EN ISO 17178 and complied with EU thresholds for inert-waste leaching under the conditions of this study, supporting their suitability for construction use.

Among the tested oils, the sunflower-oil formulation delivered the highest mechanical strength—particularly in shear performance after extended curing—and exhibited the highest calorific value. In headspace GC–MS screening, sunflower (and, to a lesser extent, linseed) showed tentatively assignable peaks (library match > 75%); no toxicological or regulatory inferences are drawn from these tentative assignments. In contrast, the rapeseed- and castor-oil formulations showed no peaks above the identification threshold in this screening (which does not imply the absence of emissions) and demonstrated excellent leaching performance, with all parameters below quantification thresholds. Both systems also maintained stable mechanical performance, especially in tensile strength after thermal aging. Although the castor-oil formulation exhibited slightly lower shear strength and increased viscosity, its low headspace signal in this screening makes it a promising option for low-emission candidates, pending chamber-based evaluation.

Rapeseed oil offered the most balanced overall profile, combining high mechanical performance, a low headspace signal in screening, and consistent environmental results. Formulations based on linseed oil and used cooking oil also met the specified requirements but showed greater variability in mechanical stability. In the case of used cooking oil, detectable levels of Zn and Sb in leachates further highlighted the importance of feedstock quality control when using recycled oils.

In conclusion, while sunflower oil may be well suited for strength-oriented formulations, rapeseed and castor oils emerge as balanced alternatives offering both functional advantages and robust leaching outcomes. These findings support the continued development of polyurethane adhesives incorporating unmodified bio-based oils. The unmodified-oil route reduces formulation complexity and avoids derivatization steps that increase processing burden and embodied carbon yet delivers EN ISO 17178–compliant performance at 10 wt% in 2K parquet adhesives.

Future work should include chamber-based VOC assessments (e.g., ISO 16000-9/EN 16516), long-term durability studies under service-like conditions, and measures to ensure quality consistency in waste-derived raw materials.

## Figures and Tables

**Table 1:** Key oil properties relevant to PU behavior. Adapted from [11].

	Rapeseed Oil	Sunflower Oil	Linseed Oil	Castor Oil	Used Cooking Oil
Properties					
Acid Value (AV) [mgKOH/g]	0.15	0.21	0.41	0.91	0.94
Hydroxyl Value (OHV) [mgKOH/g]	0.66	2.92	3.73	158.04	4.22
Iodine Value (IV) [gJ_2_/100 g]	110.6	124.1	173.2	85.5	98.0
Peroxide Value (PV) [meqO_2_/kg]	1.85	5.18	7.65	16.75	32.8
Water Content [%]	0.04	0.05	0.06	0.15	0.20
Viscosity [mPas]	71.8	63.2	55.5	900	102.7
Density [g/cm^3^]	0.916	0.910	0.928	0.955	0.918

**Table 2:** Results of leaching tests for selected elements and anions from adhesive formulations.

Element	Unit	Reference	Rapeseed Oil	Sunflower Oil	Linseed Oil	Castor Oil	Used Cooking Oil
Mercury (Hg)	mg/kg d.m.	<0.01	<0.01	<0.01	<0.01	<0.01	<0.01
Molybdenum (Mo)	mg/kg d.m.	<0.04	<0.04	<0.04	<0.04	<0.04	<0.04
Nickel (Ni)	mg/kg d.m.	<0.04	<0.04	<0.04	<0.04	<0.04	<0.04
Lead (Pb)	mg/kg d.m.	<0.1	<0.1	<0.1	<0.1	<0.1	<0.1
Selenium (Se)	mg/kg d.m.	<0.025	<0.025	<0.025	<0.025	<0.025	<0.025
Copper (Cu)	mg/kg d.m.	<0.04	<0.04	<0.04	<0.04	<0.04	<0.04
Arsenic (As)	mg/kg d.m.	<0.025	<0.025	<0.025	<0.025	<0.025	<0.025
Chromium (Cr)	mg/kg d.m.	<0.03	<0.03	<0.03	<0.03	<0.03	<0.03
Cadmium (Cd)	mg/kg d.m.	<0.005	<0.005	<0.005	<0.005	<0.005	<0.005
Chlorides (Cl^−^)	mg/kg d.m.	<50	<50	<50	<50	<50	<50
Fluorides (F^−^)	mg/kg d.m.	<1.0	<1.0	<1.0	<1.0	<1.0	<1.0
Sulfates (SO_4_^2−^)	mg/kg d.m.	<100	<100	<100	<100	<100	<100

**Table 3:** Higher heating values (HHV) of cured adhesive formulations determined via bomb calorimetry (mean of three replicates, in J/g).

Formulation	HHV (J/g)
Reference	15,803
Rapeseed oil	15,898
Sunflower oil	16,120
Linseed oil	15,895
Castor oil	15,830
Used cooking oil	15,876

## Data Availability

The original contributions presented in this study are included in the article/Supplementary Material. Further inquiries can be directed to the corresponding author.

## Supplementary Materials

The following supporting information can be downloaded at: https://www.mdpi.com/article/10.3390/molecules30183780/s1, Table S1. Fatty-acid composition (wt%) of the oils used in this study. Table S2. Physicochemical descriptors of the oils (as used in Section 2.1). Figure S1. Representative structures. (A) Triglyceride backbone. (B) Ricinoleic acid (castor) bearing a C12 secondary (side-chain) hydroxyl reactive with –NCO (urethane formation). (C) Unsaturated C18 residues without hydroxyl (oleic C18:1, linoleic C18:2, α-linolenic C18:3) typical of rapeseed/sunflower/linseed; these act as non-reactive plasticizers. UCO denotes a variable mixture that may include oxidized/thermally altered species (–OOH/–OH/epoxides/aldehydes). Scheme S1. Reaction pathways relevant to this study. (A) Urethane formation between –NCO and the ricinoleic –OH (castor oil), leading to a urethane linkage (network anchoring). (B) Moisture side-reaction: –NCO + H2O → carbamic acid → amine + CO2; the amine then reacts with –NCO to form a urea linkage. This pathway is pertinent for higher-moisture or oxidized feeds (e.g., UCO).
